# *SERPINB5 *and *AKAP12 *-- Expression and promoter methylation of metastasis suppressor genes in pancreatic ductal adenocarcinoma

**DOI:** 10.1186/1471-2407-10-549

**Published:** 2010-10-12

**Authors:** Wolf A Mardin, Kostadin O Petrov, Andreas Enns, Norbert Senninger, Joerg Haier, Soeren T Mees

**Affiliations:** 1 Dept. of General and Visceral Surgery, University Hospital of Muenster, Waldeyerstr. 1, 48149 Muenster, Germany; 2 Max Delbrueck Center for Molecular Medicine, Robert-Roessle-Str. 10, 13125 Buch, Berlin, Germany

## Abstract

**Background:**

Early metastasis and infiltration are survival limiting characteristics of pancreatic ductal adenocarcinoma (PDAC). Thus, PDAC is likely to harbor alterations in metastasis suppressor genes that may provide novel diagnostic and therapeutic opportunities. This study investigates a panel of metastasis suppressor genes in correlation to PDAC phenotype and examines promoter methylation for regulatory influence on metastasis suppressor gene expression and for its potential as a diagnostic tool.

**Methods:**

Metastatic and invasive potential of 16 PDAC cell lines were quantified in an orthotopic mouse model and mRNA expression of 11 metastasis suppressor genes determined by quantitative RT-PCR. Analysis for promoter methylation was performed using methylation specific PCR and bisulfite sequencing PCR. Protein expression was determined by Western blot.

**Results:**

In general, higher metastasis suppressor gene mRNA expression was not consistent with less aggressive phenotypes of PDAC. Instead, mRNA overexpression of several metastasis suppressor genes was found in PDAC cell lines vs. normal pancreatic RNA. Of the investigated metastasis suppressor genes, only higher *AKAP12 *mRNA expression was correlated with decreased metastasis (P < 0.05) and invasion scores (P < 0.01) while higher *SERPINB5 *mRNA expression was correlated with increased metastasis scores (P < 0.05). Both genes' promoters showed methylation, but only increased *SERPINB5 *methylation was associated with loss of mRNA and protein expression (P < 0.05). *SERPINB5 *methylation was also directly correlated to decreased metastasis scores (P < 0.05).

**Conclusions:**

*AKAP12 *mRNA expression was correlated to attenuated invasive and metastatic potential and may be associated with less aggressive phenotypes of PDAC while no such evidence was obtained for the remaining metastasis suppressor genes. Increased *SERPINB5 *mRNA expression was correlated to increased metastasis and mRNA expression was regulated by methylation. Thus, *SERPINB5 *methylation was directly correlated to metastasis scores and may provide a diagnostic tool for PDAC.

## Background

Pancreatic ductal adenocarcinoma (PDAC) represents ~95% of all pancreatic malignancies and has the poorest survival rate for any solid cancer with 5-year survival rates below 5% and a median survival of 6 months [1]. Complete surgical resection is the only curative treatment option but patients are often diagnosed at late stages when surrounding tissue has already been infiltrated and metastasis has occurred. Lack of typical clinical symptoms, unavailability of methods for early detection and resistance to chemotherapy additionally contribute to the high mortality rates. Tumor biology, clinical characteristics and therapeutic response rates suggest specific alterations in PDAC that set it apart from other types of cancer [2], emphasizing the need for specific diagnostic and therapeutic targets in this tumor entity.

Genes that can inhibit metastasis and invasion are likely to be deregulated in PDAC and could be candidates for novel therapeutic and diagnostic strategies. Metastasis suppressor genes are defined as inhibitors of metastasis at any step of the metastatic cascade that do not interfere with primary tumor growth [3]. The discovery of these endogenous molecules that can exclusively inhibit metastasis, and the understanding of their actions, suggest that metastasis is an amenable therapeutic target. However, only few of the currently known metastasis suppressor genes have been examined in pancreatic cancer so far, their expression and also their means of regulation in this tumor entity remain largely uninvestigated.

DNA methylation is a well known mechanism for loss of expression in a variety of tumors, including PDAC. Aberrant methylation appears to be a frequent epigenetic event for a number of genes in pancreatic cancer that may play a role in its tumorigenesis, local progression and metastasis [4,5]. Hypermethylation has additionally been shown to coincide with tumor dedifferentiation [6,7]. At the same time, hypomethylation resulting in overexpression of affected genes has also been reported in clinical PDAC samples and -cell lines [8].

These findings could be exploited in diagnostic strategies based upon the detection of methylation in body fluids such as serum, urine, pancreatic juice or sputum [9].

In this study, a panel of genes, representing the known metastasis suppressor genes at the time of conception of the study [10], was investigated in order to determine potential candidate genes for PDAC therapy and -detection. By searching for metastasis suppressor gene expression patterns in correlation to PDAC phenotype, relevant gene candidates were identified for further research. These candidates were subsequently investigated for promoter methylation as a regulatory element that may also serve as an instrument of PDAC detection.

## Methods

Analyses were carried out for 11 metastasis suppressor genes as reviewed by Shevde et al. [10]: *AKAP12*, *BRMS1*, *CD82*, *CDH1*, *KiSS-1*, *MAP2K4*, *MED23*, *NDRG1*, *SERPINB5*, *TIMP3 *and *TXNIP*. Alternative names and accession numbers are provided in Additional file 1.

A total of 18 human PDAC cell lines were analyzed (Table 1). Cells were maintained in recommended growth media, and all media were supplemented with 10% heat inactivated fetal bovine serum (Gibco/Invitrogen, Karlsruhe, Germany) and were mycoplasma negative. For culturing, they were incubated at 37°C in humidified air with 5% or 10% CO_2_. The medium was replaced twice a week, and cells were maintained by serial passaging after trypsinization with 0.1% trypsin.

**Table 1:** Tumor biology of PDAC cell lines

Metastasis Score	Cell Line	Origin	Invasion Score	Cell Line	Origin
0.22	Capan2	PT	0.25	PaTu 8902	PT
0.73	PaTu 8902	PT	0.56	Capan2	PT
0.75	PaTu 8988T	LM	0.94	Panc1	PT
0.90	Panc1	PT	0.98	A818	AS
0.92	BxPC3	PT	1.50	BxPC3	PT
1.24	A818	AS	2.08	PaTu 8988T	LM
1.44	PT45	PT	2.49	PT45	PT
2.16	Capan1	PT	3.27	Capan1	PT
2.58	MiaPaCa2	PT	3.04	HPAF2	AS
3.54	SU 86.86	LM	3.43	SU 86.86	LM
3.55	Suit 0028	LM	3.53	MPanc 96	PT
4.29	HPAF2	AS	3.99	PaTu 8988S	LM
4.29	MPanc 96	PT	5.60	MiaPaCa2	PT
4.38	AsPc1	AS	6.25	Suit 007	LM
4.90	PaTu 8988S	LM	6.44	AsPc1	AS
5.49	Suit 007	LM	8.82	Suit 0028	LM
N/A	HS766T	NM	N/A	HS766T	NM
N/A	PL45	PT	N/A	PL45	PT

Score-based assessment of metastatic and invasive potential. Cell line origins: PT = primary tumor, AS = ascites, LM = liver metastasis, NM = Lymph node metastasis. Cell lines PL45 and HS766T did not grow tumors in the mouse model but were included in methylation assays.

An orthotopic implantation tumor model using four-week-old male nude mice (Crl:NU/NU-nuBR) was previously performed, including calculation of metastasis and invasion scores [11]. All experiments were conducted in accordance with the national guidelines for the care and use of laboratory animals, and the experimental protocol was approved by the state agency for animal welfare of North Rhine-Westphalia (9.93.2.10.36.07.257, LANUV, NRW, Germany). In brief, Four-week-old male nude mice (Crl:NU/NU-nuBR) were used as tumor donors and received a subcutaneous injection of each human PDAC cell line. The mice were euthanized after 3 to 4 weeks and the donor tumors were collected. Donor tumor fragments were inserted orthotopically into the recipient mouse's pancreatic parenchyma. After 12 weeks of tumor growth clinical signs of tumor burden, primary tumor volume, local infiltration, and patterns of local and systemic metastases were assessed with a standardized dissemination score. Local infiltration was determined at the abdominal organs, the retroperitoneum and the abdominal wall. Metastasis was determined at liver, lymph nodes in the upper abdomen, diaphragm, mesentery and retroperitoneum; isolated tumor nodules with no anatomic connection to the primary tumor were also defined as metastases. Score values represent mean sums of the obtained credit points for all mice in the specific group/cell line (n = 10). The results were compiled into a score each for metastasis and invasion.

DNA extraction from cultured cell lines was performed with DNeasy Blood & Tissue kits (Qiagen, Hildesheim, Germany) according to the manufacturer's specifications. DNA samples were stored at -20°C. RNA extraction was performed from cultured cell lines using RNeasy Mini kits (Qiagen) according to the manufacturer's specifications. A total RNA preparation from normal human pancreas was acquired from Applied Biosystems/Applera (Darmstadt, Germany). RNA samples were stored at -80°C. CDNA was generated with High Capacity cDNA Reverse Transcription kits (Applied Biosystems) according to the manufacturer's specifications. CDNA was stored at -20°C. Purity and concentration of nucleic acids were measured in a biophotometer (Eppendorf; Hamburg, Germany). Bisulfite modification of DNA was performed with EpiTect Bisulfite kits (Qiagen) according to the manufacturer's specifications. Obtained products were stored at -20°C.

MSP Primers were designed to be located in CpG-islands within the promoter region of each gene. Primer sequences, annealing temperatures and product sizes are provided in Additional file 2. All primers were obtained from Operon (Hildesheim, Germany) and used at final concentrations of 2 μM. Methylation specific PCR (MSP) was performed as a hot-start PCR in a mastercycler gradient (Eppendorf) under the following conditions in a total reaction volume of 25 μl. The reaction was hot-started using 1 U of GoTaq (Promega, Mannheim, Germany) after 5 min at 95°C. 35 cycles were run using the following parameters: 0:30 min at T_a_, 1 min at 72°C. Final extension was allowed for 10 min at 72°C. Products were kept at 4°C until further use (maximum of 24 h). Unmethylated EpiTect Control DNA (Qiagen) served as a negative methylation control. DNA isolated from peripheral blood from healthy individuals was artificially methylated using the SSSI Methylation Kit (NEB, Ipswich, MA, USA) according to the manufacturer's specifications and used as a positive control for the methylation-specific primers after bisulfite modification. No-template controls were conducted in parallel.

BSP-Primers were designed to amplify a region containing ~20-40 CpG-dinucleotides within the CpG-islands in the promoter region of each gene. Primer sequences, annealing temperatures, number of CpGs sequenced and product sizes are provided in Additional file 3. Each primer included an additional M13 sequence which was used in the following sequencing reaction. Primers were obtained from Operon and used at final concentrations of 1.4 μM. Bisulfite sequencing PCR (BSP) was performed using 0.7 U of HotStarTaq Plus (Qiagen) per reaction. After 5 min of denaturation at 95°C, an initial 5 cycles were run with 0:30 min at T_a1_, 1 min at 72°C followed by 35 cycles with 0:30 min at T_a2_, 1 min at 72°C. Final extension was allowed for 10 min at 72°C. Products were kept at 4°C until further use for a maximum of 24 h. No-template controls were performed in parallel. PCR products were run on an agarose gel stained with ethidium bromide and the bands were excised with clean scalpels. Agarose and contaminants were removed using QiaQuick Gel Extraction kits (Qiagen) according to the manufacturer's specifications. Dye terminator sequencing reactions of the cleaned PCR products were performed with Quickstart reaction kits (Beckman Coulter; Krefeld, Germany) and M13 sequencing primers according to the manufacturer's specifications. Analysis of the sequencing reactions was performed on a Beckman Coulter 8800 Sequencer. The results from direct sequencing were analyzed with the Beckman Coulter CEQ 8800 Genetic Analysis System software v9.0 using C- to T-peak ratios to define a CpG-dinucleotide as methylated, unmethylated or heterogeneously methylated. Point values were assigned to each CpG-dinucleotide according to its methylation status as follows: unmethylated: 0; heterogeneously methylated: 1; methylated: 2. A methylation-score was calculated for each gene in each cell line by using the average point value of all investigated CpG-dinucleotides for that gene.

Western Blots were performed as previously described [11]. The following adaptations were made: 15 μg (*SERPINB5*) or 10 μg (*AKAP12*) of protein were used per cell line and lysates were loaded on 10% (*SERPINB5*) or 4% (*AKAP12*) polyacrylamid gels. *AKAP12 *was detected with 0.02 μg/ml rabbit anti-*AKAP12 *antibody (Novus Biologicals, Littleton, CO, USA) and *SERPINB5 *was detected with 0.67 μg/ml rabbit anti-*SERPINB5 *antibody (Aviva Systems Biology, San Diego, CA, USA). Investigated cell lines were selected to represent methylated and unmethylated factions.

Quantitative reverse transcriptase-PCR (qRT-PCR) was performed using TaqMan assays (Applied Biosystems) in accordance with the manufacturer's instructions. A normal human pancreatic RNA sample was acquired from Ambion/Applied Biosystems (Darmstadt, Germany). The assays used 50 ng of cDNA template per sample and PPIB and HPRT1 were used as housekeeping genes. All reverse transcriptase reactions included no-template controls and real-time minus controls. RNA expression levels were quantified using the ABI Prism 7900HT Sequence Detection System (Applied Biosystems). QRT-PCR was performed in triplicate, including no-template controls. Relative expression was calculated using the 2^-ΔΔCT ^Method and data was analyzed with Real-Time StatMiner (Integromics; Madrid, Spain) and QBase v1.3.5 [12].

Metastasis suppressor gene co-expression was evaluated using 2-tailed Pearson's correlation while correlations between metastatic/invasive potential, metastasis suppressor gene mRNA expression and promoter methylation were investigated using 2-tailed Spearman's correlation and analysis of variance (ANOVA) was conducted using the Kruskal-Wallis test. 95% confidence intervals were calculated and P < 0.05 was considered significant. SPSS 16.0 (SPSS Inc., Chicago, IL, USA) was used for these calculations.

## Results

### Animal model: Tumor biology of the PDAC cell lines

Metastasis- and invasion-scores were determined for 16 of the 18 PDAC cell lines (Table 1). Two of the 18 investigated cell lines (PL45 and HS766T) did not grow subcutaneous tumors, but mRNA expression and methylation status analyses were still carried out for these cell lines.

### Metastasis suppressor gene mRNA expression

**Figure 1:**
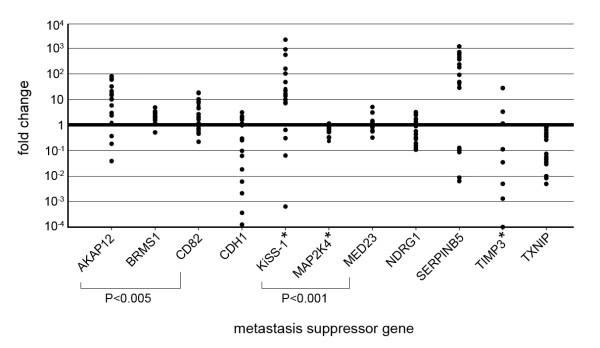
**mRNA expression of metastasis suppressor genes**. Metastasis suppressor gene expression in PDAC cell lines as fold change of expression in normal pancreatic RNA. Each dot represents a PDAC cell line. Only *MAP2K4 *and *TXNIP *show downregulation in PDAC cell lines while most other genes are upregulated. *not detected in some cell lines: *KiSS-1 *not detected in MIAPaCa-2. *MAP2K4 *not detected in A818, MPanc 96, PaTu 8902, PaTu 8988S and PaTu 8988T. *TIMP3 *not detected in A818, MIAPaCa-2, PaTu 8902 and PaTu 8988T. *AKAP12 *and *CD82 *mRNA expression correlated to one another (P < 0.005). *KiSS-1 *and *MED23 *mRNA expression correlated to one another (P < 0.001).

With the exception of *MAP2K4 *and *TXNIP*, the majority of the metastasis suppressor genes showed overexpression in the PDAC cell lines vs. the normal pancreatic RNA (Figure 1; all expression data are provided in Additional file 4).

The mRNA expression of two gene-pairs was highly positively correlated: *CD82*/*AKAP12 *(P < 0.005) and *KiSS-1*/*MED23 *(P < 0.001). The link between *KiSS-1 *and *MED23 *(CRSP3, DRIP130) has been described previously: *MED23 *is a cofactor required for expression of *KiSS-1 *[13]. A literature search failed to reveal a link between *CD82 *and *AKAP12 *and to the best of our knowledge this is the first description of their co-expression.

**Figure 2:**
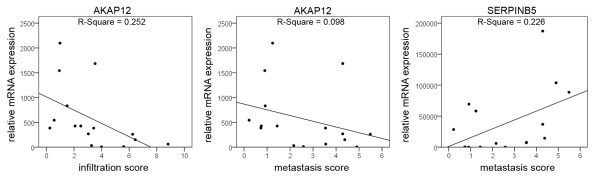
**Metastasis suppressor gene expression and tumor biology**. Only *AKAP12 *and *SERPINB5 *mRNA expression showed correlation to tumor phenotype. Increased *AKAP12 *mRNA expression was significantly correlated with reduced metastasis (P < 0.05) and invasion (P < 0.01) while *SERPINB5 *expression was correlated with increased metastasis (P < 0.05).

Significant correlations with invasion and metastasis scores were detected only for *AKAP12 *and *SERPINB5 *mRNA expression. Overexpression of *AKAP12 *was correlated with lower metastasis-scores (P < 0.05) and lower invasion scores (P < 0.005). In contrast, overexpression of *SERPINB5 *correlated with increased metastasis scores (P < 0.05) (Figure 2). Confirmation of these results by ANOVA was possible for *SERPINB5 *expression vs. metastasis score (P < 0.05) and *AKAP12 *expression vs. infiltration score (P < 0.01).

### Promoter methylation

**Figure 3:**
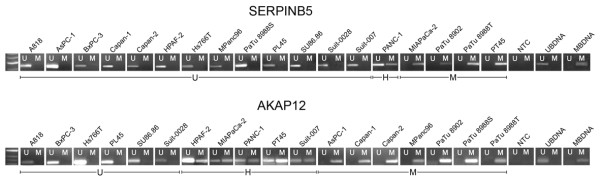
**Methylation specific PCR for *AKAP12 *and *SERPINB5***.

As *AKAP12 *and *SERPINB5 *showed correlations to invasion and metastasis scores, we proceeded to investigate the genes' promoter methylation. MSP analysis of CpG-islands in the promoter region of the investigated genes showed three different patterns. 1) Only the PCR with the methylation-specific primer resulted in a band. 2) Only the PCR with the unmethylation-specific primer had a band. 3) Both PCRs from the same MSP showed bands simultaneously. The results were therefore classified as methylated, unmethylated or heterogeneously methylated (Figure 3). MSP detected methylation in 12/18 (67%) cell lines in *AKAP12 *and 5/18 (28%) cell lines in *SERPINB5*.

**Figure 4:**
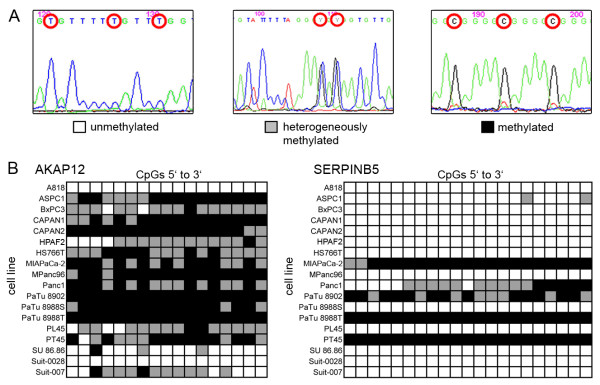
**Bisulfite sequencing**. Signals from BSP with direct sequencing. During direct sequencing cleaned up PCR products without prior cloning were used, the sequenced signal therefore represents average methylation across the entire PCR template. **A) **3 distinct signal patterns were identified: Unmethylated cytosines with a T-signal at the CpG site, heterogeneously methylated CpGs with superimposing C- and T-signals and methylated CpGs with a C-signal. **B) **BSP-analysis of promoter methylation for *AKAP12 *and *SERPINB5*. Each box represents a sequenced CpG-dinucleotide.

BSP produced three different signal types for the CpG-dinucleotides within the sequenced region: 1) Detection of a T-peak and no C-peak was classified as an unmethylated CpG-dinucleotide. 2) Presence of a C-peak and no T-peak was classified as a methylated CpG-dinucleotide. 3) Superimposing C- and T-peaks were classified as heterogeneous methylation (Figure 4a). The signals were translated into a methylation-score (Additional file 5). BSP showed methylation in 15/18 (83%) cell lines in *AKAP12 *and in 5/18 (28%) cell lines in *SERPINB5 *(Figure 4b).

MSP-status was compared to BSP methylation-score and both techniques were highly correlated across all samples (P < 0.005) showing the same results for each sample in MSP and BSP. Differing results between MSP and BSP were however obtained for three cell lines (BxPC3, HS766T, PL45) in *AKAP12 *where BSP showed methylation that was not detected by MSP.

**Figure 5:**
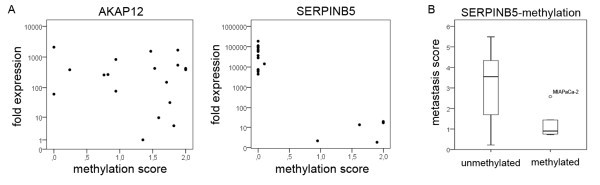
**Methylation, mRNA expression and metastasis score**. **A) **mRNA expression was plotted against methylation-score for each cell line to demonstrate the relevance of promoter methylation to mRNA expression. *AKAP12 *shows no significant correlation while *SERPINB5 *expression is significantly reduced in methylated cell lines. **B) **Cell lines with methylated *SERPINB5 *promoters have significantly lower metastatic potential (P < 0.05). MIAPaCa-2 is an outlier in the methylated group but has reduced metastatic ability compared to its strong invasive potential.

We next investigated the influence of promoter methylation on mRNA expression. For *SERPINB5 *the methylated cell lines had the lowest mRNA expression across all cell lines while no significant influence on mRNA expression was detected for *AKAP12 *(Figure 5a).

As *SERPINB5 *expression was correlated both to methylation and metastatic potential, we investigated whether *SERPINB5 *methylation was directly correlated to metastatic potential. Spearman correlation revealed that *SERPINB5*-methylated cell lines had significantly lower metastasis scores than unmethylated cell lines (P < 0.05) (Figure 5b).

### Protein expression

**Figure 6:**
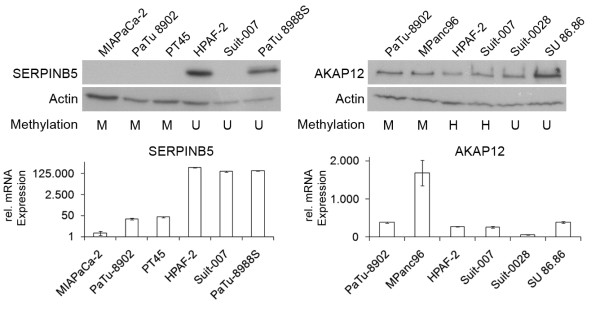
**Protein expression of *AKAP12 *and *SERPINB5***. Western Blot, methylation status and mRNA expression of selected cell lines. *SERPINB5 *protein is lost in methylated cell lines along with mRNA expression while *AKAP12 *protein remains present in methylated cell lines.

Western Blot showed *AKAP12 *protein to be present in all investigated cell lines regardless of their methylation status and mRNA expression, making it an unlikely candidate for methylation based regulation. In contrast, *SERPINB5 *protein was not present in methylated cell lines which all exhibited low mRNA expression. However, *SERPINB5 *protein was also not detected in Suit-007, which was not methylated for the gene and had high *SERPINB5 *mRNA expression, suggesting an alternative mode of regulation (Figure 6). Nonetheless, our data is in accord with reports of *SERPINB5 *in the literature, confirming its repression by promoter methylation on the protein level.

## Discussion

PDAC is set apart from many other tumor entities by specific genetic alterations, a strong desmoplastic reaction, early metastasis and early infiltration of neighboring organs. Its response rates to chemotherapy are dismal and survival rates remain low despite modern interdisciplinary approaches. In this study, we have investigated a panel of genes involved in key processes of tumor dissemination in an attempt to identify promising candidates for novel therapeutic and diagnostic strategies. Metastasis suppressor gene mRNA expression was correlated with highly varying invasive and metastatic phenotypes of human PDAC cell lines in an orthotopic mouse model. Overexpression of the investigated metastasis suppressors was found in most tumor cell lines compared to normal pancreatic RNA and, in contrast to other tumor entities, only one of the genes in the investigated panel, *AKAP12*, showed a correlation between increased mRNA expression and diminished invasive and metastatic capabilities. *AKAP12 *was overexpressed vs. the normal pancreatic RNA sample in 83% (15/18) of PDAC cell lines and this overexpression has also been reported in patient samples [14]. While several tumor suppressor genes were overexpressed in the PDAC cell lines vs. a control sample of normal pancreatic RNA, it is important to note that the composition of the control sample is not ideal. Normal pancreas includes a variety of cell types and thus is not an accurate control for the expression levels in normal ductal cells. Microdissection of ductal pancreatic cells from normal tissue could provide a more specific profile that may better correspond to the PDAC cell lines. However, the differences in sample preparation and nucleic acid isolation would have a relevant influence on the constitution of the specimens.

While some authors observed a correlation between loss of mRNA expression and promoter methylation for *AKAP12 *in other cancers [15], we did not find significant evidence that this also takes place in the investigated PDAC cell lines. Loss of protein expression also failed to match promoter methylation as well as reduced mRNA expression in the investigated cell lines. Interestingly, we detected a correlation between increased *SERPINB5 *(*MASPIN*) mRNA expression and increased propensity to metastasize. *SERPINB5 *is regarded as a metastasis suppressor gene in breast cancer [16], but has also been proposed as a detector of circulating breast cancer cells [17]. It is involved in intestinal cancer where its overexpression appears to coincide with tumor progression and metastatic spread [18,19]. For pancreatic cancer, conflicting roles of *SERPINB5 *have been described: Hong et al. showed *SERPINB5*-transfected PDAC cells to have reduced invasive ability [20] and Kashima et al. found *SERPINB5 *expression to increase with tumor progression from intraductal papillary mucinous neoplasm to non-invasive carcinomas but to decrease in invasive carcinomas [21]. In contrast to these reports of protective effects, overexpression of *SERPINB5 *in PDAC was found to be associated with worse postoperative survival and was an independent adverse prognosticator for postoperative survival [22-24]. Previous studies have suggested that *SERPINB5 *must be located at the cytoplasmic membrane in order to perform its protective role, but the details of its function still remain unclear [16]. *SERPINB5 *is not expressed in healthy pancreas and its expression is controlled by promoter methylation [25,26]. Accordingly, in this study, *SERPINB5*-methylated cell lines had more than 100-fold reduced mRNA expression vs. unmethylated cell lines and *SERPINB5 *methylation corresponded to loss of protein expression. Additionally, a significant correlation between *SERPINB5 *methylation levels and PDAC cell line metastatic potential was detected. Thus, an assay determining *SERPINB5 *promoter methylation, or rather lack of methylation, may be considered as a marker for pancreatic cancer. Functional analyses of the role of *SERPINB5 *in PDAC and validation of its overexpression using resected specimens are necessary before any conclusions regarding its usefulness as a diagnostic or therapeutic option can be reached; this is the subject of ongoing studies. However, the results of the present study may be used to identify candidate genes for subsequent studies.

## Conclusions

Our data suggests that of 11 investigated metastasis suppressors only *AKAP12 *may be associated with a less aggressive phenotype of PDAC, while *SERPINB5 *expression may even correspond to invasive tumors. In contrast to other tumor entities, metastasis suppressor gene deregulation appears to be limited to a small number of known genes. These results underline the individual tumor biology of PDAC and further emphasize the need for specific markers and novel therapeutic targets for this entity.

## Competing interests

The authors declare that they have no competing interests.

## Authors' contributions

All authors read and approved the final manuscript.

WAM carried out molecular genetic studies, participated in the design and coordination of the study and drafted the manuscript. KP helped with the molecular genetic studies, participated in the design of the study and helped draft the manuscript. AE helped with the molecular genetic studies and participated in the design of the study. NS participated in the study design and coordination and helped to draft the manuscript. JH conceived the study, and participated in its design and coordination and helped to draft the manuscript. STM carried out the animal model, participated in the study design and coordination and helped to draft the manuscript.

## Pre-publication history

The pre-publication history for this paper can be accessed here:

http://www.biomedcentral.com/1471-2407/10/549/prepub

## Supplementary Material

Additional file 1 **Gene names**. File contains alternative gene names and RefSeqs for the investigated genes. Click here for file

Additional file 2 **MSP primer details**. File contains sequences, product size and annealing temperatures for the primers used in MSP. Click here for file

Additional file 3 **BSP primer details**. File contains sequences, product size, number of sequenced CpG sites and annealing temperatures for the primers used in BSP. Click here for file

Additional file 4 **qRT-PCR data**. File contains the relative expression of the investigated genes and reference genes as determined by qRT-PCR. Click here for file

Additional file 5 **Methylation score**. File contains the values of the methylation score and -rank that were calculated for each gene and cell line based on the results of BSP. Click here for file
