# Supplementary material for: SERPINB5 and AKAP12 -- Expression and promoter methylation of metastasis suppressor genes in pancreatic ductal adenocarcinoma
Source: BMC Cancer. 2010 Oct 12;10:549. doi: 10.1186/1471-2407-10-549 (PMC2966466; doi:10.1186/1471-2407-10-549)
Supplement: Additional file 1 — Gene names. File contains alternative gene names and RefSeqs for the investigated genes. [file 1471-2407-10-549-S1.PDF]

Gene names and RefSeqs

Gene		Alias	RefSeq
AKAP12	A Kinase (PRKA) Anchor Protein (Gravin) 12	AKAP250<br>DKFZp686M0430<br>DKFZp686O0331<br>FLJ20945<br>FLJ97621<br>SSeCKS (rodent)	NM_144497.1<br>NM_005100.2
BRMS1	Breast Cancer Metastasis Suppressor 1	DKFZp564A063	NM_015399.3<br>NM_001024957.1
CD82	CD82 Molecule	4F9<br>C33<br>GR15<br>IA4<br>KAI1<br>R2<br>SAR2<br>ST6<br>TSPAN27	NM_001024844.1<br>NM_002231.3
CDH1	Cadherin 1, Type 1, E-Cadherin (Epithelial)	Arc-1<br>CD324<br>CDHE<br>ECAD<br>LCAM<br>UVO	NM_004360.2
KiSS-1	KiSS-1 Metastasis-Suppressor	KiSS-1<br>METASTIN<br>MGC39258	NM_002256.3
MAP2K4	Mitogen-Activated Protein Kinase Kinase 4	JNKK<br>JNKK1<br>MAPKK4<br>MEK4<br>MKK4<br>PRKMK4<br>SEK1<br>SERK1	NM_003010.2
MED23	Cofactor required for Sp1 Transcriptional Activation, Subunit 3, 130kDah	CRSP3<br>CRSP130<br>CRSP133<br>DKFZp434H0117<br>DRIP130<br>RP5-914N13.2<br>SUR2	NM_004830.2
NDRG1	N-myc Downstream Regulated Gene 1	CAP43<br>CMT4D<br>DRG1<br>GC4<br>HMSNL<br>NDR1<br>NMSL<br>PROXY1<br>RIT42<br>RTP<br>TARG1<br>TDD5	NM_006096.2
SERPINB5	Serpin Peptidase Inhibitor, Clade B (Ovalbumin), Member 5	PI5<br>MASPIN	NM_002639.2
TIMP3	TIMP Metallopeptidase Inhibitor 3 (Sorsby Fundus Dystrophy, Pseudoinflammatory)	HSMRK222<br>K222<br>K222TA2<br>SFD	NM_000362.4
TXNIP	Thioredoxin Interacting Protein	EST01027<br>HHCPA78<br>THIF<br>VDUP1	NM_006472.1
