# Supplementary material for: SERPINB5 and AKAP12 -- Expression and promoter methylation of metastasis suppressor genes in pancreatic ductal adenocarcinoma
Source: BMC Cancer. 2010 Oct 12;10:549. doi: 10.1186/1471-2407-10-549 (PMC2966466; doi:10.1186/1471-2407-10-549)
Supplement: Additional file 2 — MSP primer details. File contains sequences, product size and annealing temperatures for the primers used in MSP. [file 1471-2407-10-549-S2.PDF]

MSP Primers

Gene	Specificity	Forward Primer 5‘-3‘	Reverse Primer 5‘-3‘	Product / bp	T_A / °C
AKAP12	U	TTGGGTTGTTTTTGTAGTTTTAGTTG	AACCAAAAACACTACAACACACC	107	55
	M	GGGTCGTTTTCGTAGTTTTAGTCG	CAAAAACGCTACGACGCGCC	102	63
BRMS1	U	GATTGGGGTTGGTTTTTTTTTTTGTATGTT	CCAAAACATACTAAAATACCACAAACCAA	167	56
	M	CGGGGTCGGTTTTTTTTTTTGTACGTC	GAAACATACTAAAATACCGCGAACCGA	162	61
CD82	U	GAAGATATGTTTTTTTTGTTGGTTTTGTTTTT	AATAAAAAAAAAACTCCATAACAAAACAAAAC	161	52
	M	ATACGTTTTTTTCGTCGGTTTCGTTTTC	AAAAAAAAAACTCCGTAACGAAACGAAAC	154	56
CDH1	U	TAATTTTAGGTAGAGGGTTATTGT	CACAACCAATCAACAACACA	97	50
	M	TTAGGTTAGAGGGTTATCGCGT	TAACTAAAAATTCACCTACCGAC	115	58
KiSS-1	U	TTTAGGGTGTGGTGTTGGTGTAGTGG	CCACCCCCACCCCACATACTCTA	193	60
	M	AGGGCGCGGTGTTGGTGTAGC	GCCCCCGCCCCGCATACT	188	59
MAP2K4	U	GTTTGTTTTATAGGTTGTGTATTTAGAGTTGGG	CCACAACAACTACAATACAACAAACTCAAC	195	56
	M	GTTTTATAGGTCGCGTATTTAGAGTCGG	GCGACGACTACAATACAACAAACTCG	188	56
MED23	U	GATGGGAAGGGTTTGGTATGTGTTGT	CAACCAAAAAATAACAACCTACAATCAACA	177	56
	M	GGGAAGGGTTCGGTACGCGTCGT	CGAAAAATAACGACCTACGATCGACG	170	64
NDRG1	U	GGGTATATGTGTTTGTTGTATAGGTT	CCCAAACACACCAAACTAAACTAAA	184	53
	M	TATACGCGTTCGTTGTATAGGTC	GAACGCGCCGAACTAAACTAAA	178	53
SERPINB5	U	TGTTAGTTTAGTTTTATGTTTTGTTTTGTTTT	ATTTCACCTTCCAATCCTACATAAACCA	238	56
	M	TAGTTTAGTTTTACGTTTCGTTTCGTTTCGTTTC	TCACCTTCCGATCCTACGTAAACCG	232	56
TIMP3	U	TTTTGTTTTGTTATTTTTTGTTTTTGGTTTT	CCCCCAAAAACCCCACCTCA	122	54
	M	CGTTTCGTTATTTTTTGTTTTCGGTTTC	CCGAAAACCCCGCCTCG	116	58
TXNIP	U	TTGTGTTTATGTGTTATAGTGATTTTATTGATT	CCCAAACCAAAAAAACCACACAAAC	137	53
	M	TCGTGTTTACGCGTTATAGCGATTT	CGAACCAAAAAAACCACGCGAA	135	53
