# Supplementary material for: SERPINB5 and AKAP12 -- Expression and promoter methylation of metastasis suppressor genes in pancreatic ductal adenocarcinoma
Source: BMC Cancer. 2010 Oct 12;10:549. doi: 10.1186/1471-2407-10-549 (PMC2966466; doi:10.1186/1471-2407-10-549)
Supplement: Additional file 3 — BSP primer details. File contains sequences, product size, number of sequenced CpG sites and annealing temperatures for the primers used in BSP. [file 1471-2407-10-549-S3.PDF]

BSP Primers

Gene	Forward Primer 5‘-3‘	Reverse Primer 5‘-3‘	Product / bp	# of CpGs sequenced	T_A1 / °C	T_A2 / °C
M13 Sequencing Primer	GTAAAACGACGGCCAG	CAGGAAACAGCTATGAC	/	/	/	/
AKAP12	TGTTTTTTGAGGTTTTGGGT	AACCACCTCTTAACCTCC	228	17	46	64
BRMS1	TATTTTTTTGAGTTGGGGGTG	ACTCACAATTAAACAATTTAATCCC	215	16	49	64
CD82	TTTGGTAAGGATTTAATTAATGGTA	AACCCAACTAAAACTAAAACCCC	495	34	48	64
CDH1	TTTAGTAATTTTAGGTTAGAGGGTTAT	AAACTCACAAATACTTTACAATTCC	221	17	49	64
KISS1	GAGGAAGTTAGTTGTTATTGTTAGGT	CTCCTTTAAAATCTAAAATTCACTACC	294	24	52	65
MAP2K4	ATTTTTATGGGAATGATGAAAGTTG	CCATTATTAAAAATAAAAAACC	341	32	42	60
MED23	TGTTTTTGGGTTTTAAATTTTTTATTTTTTA	CCAACCAACAACAAAAACCTATAC	277	24	48	62
NDRG1	GAGATATAAGGAAGTTTATATGTA	TTCAACACCAACTAAAAACCAAA	423	48	48	63
SERPINB5	GTGTTTGAGAAATTTGTAGTGTTATTAT	CTACCCCACCTTACTTACCTAA	447	21	51	65
TIMP3	GTTTGGGTTAGAGATATTTAGTGGTTTAG	CCCCCTCAAACCAATAACAAAACC	271	24	48	62
TXNIP	GTTAATGGGAGGGATGTGTA	ACCCCTTTACAAAAAATTATTTCACT	349	15	50	64
