# Supplementary material for: SERPINB5 and AKAP12 -- Expression and promoter methylation of metastasis suppressor genes in pancreatic ductal adenocarcinoma
Source: BMC Cancer. 2010 Oct 12;10:549. doi: 10.1186/1471-2407-10-549 (PMC2966466; doi:10.1186/1471-2407-10-549)
Supplement: Additional file 4 — qRT-PCR data. File contains the relative expression of the investigated genes and reference genes as determined by qRT-PCR. [file 1471-2407-10-549-S4.PDF]

mRNA expression by qRT-PCR

0 = not detected

	AKAP12	1 StdDev	BRMS1	1 StdDev	CD82	1 StdDev	CDH1	1 StdDev	KISS1	1 SEM	MAP2K4	1 StdDev
**Normal_RNA**	26.12	4.28	1.98	0.27	4.56	0.64	8009.42	833.45	15.81	5.06	4.38	0.53
**A818**	2096.97	254.43	5.74	0.47	84.51	9.64	1962.91	171.10	114.72	11.76	0.00	0.00
**ASPC1**	149.70	3.40	3.95	0.10	11.89	1.11	24658.35	2873.34	344.93	36.04	3.40	0.09
**BxPC3**	832.49	58.00	4.25	0.10	11.72	0.38	15276.17	1507.28	750.46	50.24	1.43	0.07
**CAPAN1**	31.78	1.46	2.73	0.08	25.96	1.69	7365.53	658.03	403.98	9.49	1.00	0.06
**CAPAN2**	542.33	46.57	2.08	0.06	4.08	0.16	12743.54	1537.73	153.01	10.94	2.43	0.08
**HPAF-2**	268.58	6.54	3.04	0.08	34.06	1.55	17269.28	1746.03	213.63	22.14	4.21	0.15
**HS766T**	1.00	0.20	4.02	0.13	37.34	1.73	52.03	7.15	8797.36	966.28	2.29	0.09
**MiaPaCa2**	9.56	1.02	3.10	0.10	2.74	0.16	1.00	0.28	0.00	0.00	2.86	0.13
**MPanc96**	1684.74	337.50	5.82	1.06	79.48	16.11	2051.63	380.07	120.39	11.81	0.00	0.00
**Panc1**	1542.56	148.87	4.55	0.16	2.13	0.13	489.53	19.01	1.00	0.51	3.14	0.10
**PaTu-8902**	384.88	17.03	5.49	0.19	1.00	0.08	16.84	3.01	13906.17	1866.05	0.00	0.00
**PaTu-8988S**	4.76	0.62	6.65	0.45	7.59	0.24	9009.41	715.69	111.76	17.37	0.00	0.00
**PaTu-8988T**	426.11	123.89	6.52	0.76	2.20	0.45	144.35	21.02	34004.03	6135.75	0.00	0.00
**PL45**	76.24	4.90	3.65	0.14	5.18	0.24	12845.14	1224.54	258.44	35.56	2.84	0.11
**PT45**	426.00	29.99	1.00	0.25	2.05	0.10	2.88	0.77	1614.08	146.80	3.67	0.61
**SU8686**	383.64	19.63	2.26	0.05	20.56	1.04	15539.90	1674.83	2502.14	220.38	4.88	0.20
**Suit-0028**	60.91	2.40	3.19	0.19	3.23	0.12	2336.58	167.06	9.97	1.44	1.38	0.07
**Suit-007**	260.02	26.13	9.50	0.97	9.20	0.91	767.64	72.62	4.76	0.99	4.91	0.48

mRNA expression by qRT-PCR

0 = not detected

	MED23	1 StdDev	NDRG1	1 StdDev	SERPINB5	1 StdDev	TIMP3	1 StdDev	TXNIP	1 StdDev
**Normal_RNA**	3.17	0.38	9.38		158.53	33.11	38620.38	5843.62	202.05	4.56
**A818**	3.89	0.32	5.31	0.43	58293.34	10289.81	0.00	0.00	52.47	24.19
**ASPC1**	3.56	0.08	8.72	0.38	14359.75	2461.76	43579.58	7890.56	14.91	0.45
**BxPC3**	3.32	0.12	29.63	1.84	69372.38	9100.50	126349.64	28312.58	158.19	11.73
**CAPAN1**	2.92	0.10	11.46	0.55	5927.10	802.19	2.84	0.53	86.18	6.42
**CAPAN2**	1.75	0.30	2.62	0.12	28410.64	3334.63	5.10	1.68	71.76	5.40
**HPAF-2**	2.87	0.08	10.65	0.33	187160.16	28633.53	4322.75	1986.88	98.96	5.64
**HS766T**	2.55	0.12	8.82	2.99	111815.04	14887.15	1068434.60	294476.59	85.14	3.43
**MiaPaCa2**	3.38	0.12	1.46	0.10	1.00	0.76	0.00	0.00	125.39	9.71
**MPanc96**	4.18	0.77	12.55	2.63	36793.54	7552.68	1.78	0.51	67.71	23.96
**Panc1**	3.69	0.16	1.81	0.09	1.35	0.87	48.83	3.32	1.00	0.12
**PaTu-8902**	9.67	0.45	16.15	1.08	13.50	4.72	0.00	0.00	1.65	0.16
**PaTu-8988S**	3.48	0.13	2.99	0.14	103715.32	15126.69	3.32	0.56	9.59	0.49
**PaTu-8988T**	15.81	1.99	22.75	2.82	17.15	4.53	0.00	0.00	1.98	0.31
**PL45**	1.87	0.08	26.52	1.63	4461.29	431.39	1.00	0.33	11.54	0.69
**PT45**	2.81	0.09	8.38	0.98	19.99	4.42	5.60	3.81	53.42	2.92
**SU8686**	3.16	0.08	1.21	0.07	7087.72	478.98	4290.14	459.08	5.87	0.25
**Suit-0028**	1.00	0.09	1.00	0.06	7567.99	561.38	1322.13	180.30	7.09	0.33
**Suit-007**	4.55	0.46	3.98	0.39	88528.38	15570.00	190.07	20.27	8.81	2.02

mRNA expression by qRT-PCR - Housekeeping Genes

0 = not detected

	HPRT1	1 StdDev	PPIB	1 StdDev
Normal_RNA	1.00	0.17	2.65	0.46
A818	2.42	0.21	1.09	0.19
ASPC1	1.70	0.06	1.56	0.06
BxPC3	1.11	0.04	2.39	0.11
CAPAN1	1.25	0.03	2.11	0.08
CAPAN2	1.42	0.05	1.87	0.08
HPAF-2	2.33	0.06	1.14	0.03
HS766T	1.41	0.05	1.87	0.10
MiaPaCa2	2.65	0.14	1.00	0.06
MPanc96	2.41	0.97	1.10	0.20
Panc1	1.94	0.08	1.37	0.06
PaTu-8902	1.89	0.05	1.40	0.05
PaTu-8988S	1.05	0.05	2.53	0.10
PaTu-8988T	2.06	0.24	1.29	0.33
PL45	1.14	0.07	2.33	0.12
PT45	1.41	0.05	1.87	0.09
SU8686	1.03	0.02	2.57	0.09
Suit-0028	2.07	0.17	1.28	0.06
Suit-007	1.68	0.34	1.57	0.16
