# Supplementary material for: SERPINB5 and AKAP12 -- Expression and promoter methylation of metastasis suppressor genes in pancreatic ductal adenocarcinoma
Source: BMC Cancer. 2010 Oct 12;10:549. doi: 10.1186/1471-2407-10-549 (PMC2966466; doi:10.1186/1471-2407-10-549)
Supplement: Additional file 5 — Methylation score. File contains the values of the methylation score and -rank that were calculated for each gene and cell line based on the results of BSP. [file 1471-2407-10-549-S5.PDF]

The results from direct sequencing were analyzed with the Beckman Coulter CEQ 8800 Genetic Analysis System software v9.0 using C- to T-peak ratios to define a CpG-dinucleotide as methylated, unmethylated or heterogeneously methylated for each CpG-dinucleotide within the covered region. Point values were assigned to each CpG-dinucleotide according to its methylation status as follows: unmethylated: 0; heterogeneously methylated: 1; methylated: 2. A methylation-score was calculated for each gene in each cell line by using the average point value of all investigated CpG-dinucleotides for that gene. Additionally, a hierarchical methylation-rank from 0 to 4 was assigned based on the 5 following methylation-score intervals: 0 to <0.4; 0.4 to <0.8; 0.8 to <1.2; 1.2 to <1.6; 1.6 to 2.0.

Methylation-score

	**Genes**										
**Cell lines**	AKAP12	BRMS1	CD82	CDH1	KiSS-1	MAP2K4	MED23	NDRG1	SERPINB5	TIMP3	TXNIP
A818	0.000	0.000	0.000	0.000	1.875	0.000	0.000	0.000	0.000	0.167	0.000
ASPC1	1.706	0.000	0.000	0.000	0.292	0.000	0.000	0.000	0.095	1.542	0.000
BxPC3	0.941	0.000	0.000	0.000	0.083	0.000	0.000	0.000	0.000	0.000	0.000
CAPAN1	1.765	0.000	0.000	0.000	0.625	0.063	0.083	0.000	0.000	1.250	0.000
CAPAN2	1.882	0.000	0.000	0.000	0.917	0.000	0.000	0.000	0.000	0.417	0.000
HPAF2	0.824	0.125	0.000	0.000	0.167	0.000	0.000	0.021	0.000	1.000	0.000
HS766T	1.353	0.000	0.000	0.000	0.792	0.000	0.000	0.000	0.000	0.875	0.000
MiaPaCa2	1.588	0.125	0.000	1.941	1.958	0.031	0.000	0.000	1.905	1.792	0.000
MPanc96	1.882	0.125	0.000	0.059	0.000	0.000	0.042	0.000	0.000	1.583	0.000
Panc1	1.471	0.125	0.000	0.059	0.917	0.031	0.042	0.000	0.952	0.000	0.000
PaTu 8902	2.000	0.125	0.000	0.000	1.792	0.000	0.000	0.000	1.619	1.500	0.000
PaTu 8988S	1.824	0.125	0.000	0.000	1.792	0.000	0.000	0.000	0.000	1.583	0.000
PaTu 8988T	2.000	0.250	0.000	0.059	1.917	0.000	0.000	0.000	2.000	1.583	0.000
PL45	0.941	0.125	0.000	0.000	1.042	0.000	0.000	0.000	0.000	1.417	0.000
PT45	1.529	0.000	0.000	1.882	1.958	0.000	0.042	0.021	2.000	1.208	0.000
SU 86.86	0.235	0.000	0.000	0.000	0.000	0.000	0.042	0.000	0.000	0.333	0.000
Suit-0028	0.000	0.125	0.000	0.059	0.875	0.000	0.000	0.000	0.000	0.583	0.000
Suit-007	0.765	0.000	0.029	0.000	1.000	0.000	0.000	0.000	0.000	0.958	0.000

Methylation-rank

Cell lines	Genes										
	AKAP12	BRMS1	CD82	CDH1	KiSS-1	MAP2K4	MED23	NDRG1	SERPINB5	TIMP3	TXNIP
A818	0	0	0	0	4	0	0	0	0	0	0
ASPC1	4	0	0	0	0	0	0	0	0	3	0
BxPC3	2	0	0	0	0	0	0	0	0	0	0
CAPAN1	4	0	0	0	1	0	0	0	0	3	0
CAPAN2	4	0	0	0	2	0	0	0	0	1	0
HPAF2	2	0	0	0	0	0	0	0	0	2	0
HS766T	3	0	0	0	1	0	0	0	0	2	0
MiaPaCa2	3	0	0	4	4	0	0	0	4	4	0
MPanc96	4	0	0	0	0	0	0	0	0	3	0
Panc1	3	0	0	0	2	0	0	0	2	0	0
PaTu 8902	4	0	0	0	4	0	0	0	4	3	0
PaTu 8988S	4	0	0	0	4	0	0	0	0	3	0
PaTu 8988T	4	0	0	0	4	0	0	0	4	3	0
PL45	2	0	0	0	2	0	0	0	0	3	0
PT45	3	0	0	4	4	0	0	0	4	3	0
SU 86.86	0	0	0	0	0	0	0	0	0	0	0
Suit-0028	0	0	0	0	2	0	0	0	0	1	0
Suit-007	1	0	0	0	2	0	0	0	0	2	0
